# High–Pressure Processing vs. Thermal Treatment: Effect on the Stability of Polyphenols in Strawberry and Apple Products

**DOI:** 10.3390/foods10122919

**Published:** 2021-11-25

**Authors:** Gabriela Lorena Salazar-Orbea, Rocío García-Villalba, Francisco A. Tomás-Barberán, Luis Manuel Sánchez-Siles

**Affiliations:** 1 Quality, Safety and Bioactivity of Plant-Derived Foods, Centro de Edafología y Biología Aplicada del Segura-Consejo Superior de Investigaciones Científicas (CEBAS-CSIC), 30100 Murcia, Spain; glsalazar@cebas.csic.es (G.L.S.-O.); rgvillalba@cebas.csic.es (R.G.-V.); 2 Research and Nutrition Department, Hero Group, 30820 Alcantarilla, Spain; luisma.sanchez@hero.es; 3 Institute for Research and Nutrition, Hero Group, 5600 Lenzburg, Switzerland

**Keywords:** polyphenols, apple, strawberry, bioactive compounds, food processing, high-pressure processing, thermal processing, stability, storage

## Abstract

Polyphenols are important bioactive compounds that are affected by processing. The consumer’s demand for minimally processed products contributes to the increase in non-thermal technologies such as high-pressure processing (HPP) in the food industry. This review is aimed at critically discussing the positive and negative effects of thermal treatment (TT) and HPP on the stability of different polyphenol families in agro-food products obtained from strawberry and apple, two of the most used fruits in food processing. Our findings show that the phenolic content was affected by processing, fruit type, polyphenol family, and storage conditions (time and temperature) of the final product. To increase shelf life, manufacturers aiming to preserve the natural content of polyphenols need to find the sweet spot between polyphenol stability and product shelf-life since the residual enzyme activity from HPP can affect polyphenols negatively.

## 1. Introduction

**Figure 1:**
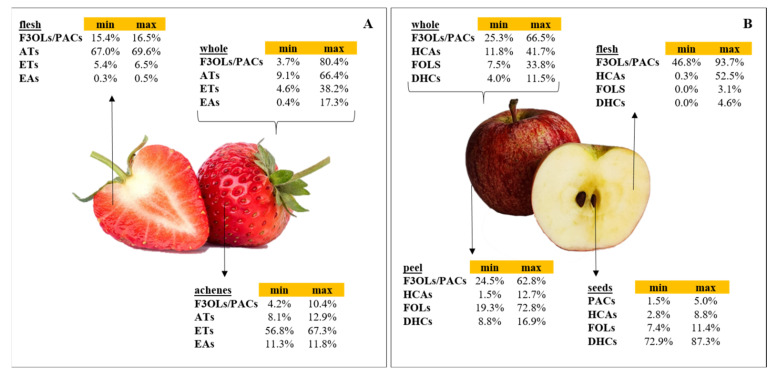
Percentage contribution of the main phenol families to the total polyphenols in different tissues of strawberry (**A**) and apple (**B**). (F3OLs/PACs) flavan-3-ols/proanthocyanidins, (ATs) anthocyanins, (ETs) ellagitannins, (EAs) ellagic acid and conjugates, (HCAs) hydroxycinnamic acids, (FOLs) flavonols, (DHCs) dihydrochalcones, (PACs) proanthocyanidins. Data retrieved from [3,4,5,6,8,9,10,11,12,13].

Strawberries and apples are fruits consumed worldwide, both fresh and processed (juices, jams, purees, smoothies, dried), due to their desirable sensory characteristics, nutritional value, and bioactive compounds. The main bioactive compounds found are polyphenols, the most significant dietary antioxidants present in fruit and vegetables, with a wide variety of biological activities linked to the associated health benefits [1]. These include flavonoid (flavonols, flavan-3-ols and proanthocyanidins, anthocyanins, dihydrochalcones, among others) and non-flavonoid (ellagitannins, ellagic acid, hydroxycinnamic acid derivatives, gallotannins) compounds. They are responsible for some of their organoleptic properties (color, aroma, astringency, bitterness) and also have relevant effects on nutrition and human health (antioxidants, anti-inflammatory, modulators of gut microbiota, etc.) [2]. There is considerable variability among the phenolic compounds found in strawberry and apple products (Figure 1), which depends on the inherent fruit characteristics (e.g., origin, variety, ripeness degree) as well as the type of food processing, and the part of the fruit (e.g., flesh, peel, achenes) [3,4].

In particular, in strawberries, flesh and achenes have a different polyphenol profile. In the flesh, the major phenols are flavan-3-ols/proanthocyanidins (F3OLs/PACs) and anthocyanins (ATs), while in achenes, the highest concentrations are for ellagitannins (ETs) and ellagic acid (EA) [3,5,6]. Similarly, in apples, there are differences in the phenolic compounds derived from flesh, peel, and seeds. In apple flesh, the major phenols are F3OLs/PACs and hydroxycinnamic acids (HCAs), being the latter mainly represented by chlorogenic acid. The principal polyphenols in apple peel are F3OLs/PACs having even higher concentrations than flesh, followed by flavonols (FOLs). In contrast, apple seeds are a more significant source of dihydrochalcones (DHCs) [4,7,8].

There is growing consumer awareness of the importance of consuming food products that preserve nutritional and functional properties [14]. Consumers’ concern for food processing is increasing in many dimensions: from its link to healthiness and naturalness to its impact on the environment [15]. Nevertheless, preservation techniques are required to retain the nutritional value, ensure food safety during shelf life, and maintain its organoleptic characteristics. A wide variety of industrial thermal and non-thermal treatment technologies are available for food preservation. Thermal treatments include conventional techniques such as pasteurization and sterilization and new thermal technologies such as ohmic heating and dielectric heating (radiofrequency and microwave).

Meanwhile, non-thermal treatments include high-pressure processing, dehydration, freezing, pulsed electric fields, cold plasma, ultrasound processing, magnetic field, and ozone [16,17]. Thermal treatment is the most widely used preservation technique. According to the intensity of heat treatment, thermal treatments may be differentiated into pasteurization (70–80 °C), sterilization (110–120 °C), and ultra-high-temperature treatment (140–160 °C) [18]. Although thermal treatments produce some adverse effects, such as loss of some nutrients (vitamin C), formation of undesirable compounds (acrylamide, heterocyclic amines, sulfur compounds), and in some cases the degradation of organoleptic characteristics, it also exerts many beneficial effects such as inactivation of foodborne pathogens, inactivation of toxins and enzymes polyphenoloxidase (PPO), peroxidase (POD), pectin-methyl esterase, improvement of digestibility and bioavailability of nutrients, improvement of some sensory characteristics (texture and flavor), enhancement of extraction of health beneficial compounds and extension of shelf life [19,20]. Strawberry and apple polyphenol oxidases and peroxidases are responsible for the oxidation of phenolic compounds when fruit tissues are disrupted in the presence of oxygen, leading to the formation of brown polymers and other discolorations that affect the organoleptic properties of the fruit and derived products negatively.

Furthermore, one of the most studied non-thermal treatments is HPP. It works with high hydrostatic pressure and is commonly used at the industrial level between 300 and 600 MPa for 3–5 min [21,22]. As a consequence of pressure, there may be some changes in physical properties (solubility, density, viscosity), kinetic reactions (acceleration or delay of reactions rate), as well as, some equilibrium processes (dissociation of weak acids, acid-base equilibria, and ionization) [23]. The effect of pressure on food components depends on the type of bond between their molecules and the interatomic distance. In general, the high-pressure treatment minimally affects compounds with covalent bonds (vitamins, minerals, folates, antioxidants, anthocyanins, and flavor compounds) [17,24]. A disadvantage of HPP is that it is not entirely effective in polyphenol oxidase, peroxidase, and pectin methylesterase inactivation at the industrial processing conditions and subsequent storage [25]. Therefore, it triggers oxidation reactions that degrade phenolic compounds and shorten the shelf life [26,27].

Briefly, given the above facts and considerations summarized as follows: (1) polyphenols are important bioactive compounds that are affected by processing, (2) consumers are increasingly demanding “minimally processed” products, and (3) several non-thermal processing technologies such as HPP are now prevalent on the market, here, the objective of this article is to conduct a review of the literature about the differences between TT and HPP on the impact in polyphenol degradation in strawberry and apple products, two of the most used fruits in food processing.

## 2. Overview of the Studies Included in the Review

We systematically searched, evaluated, and synthesized research articles on the effects of traditional TT and HPP and their subsequent storage on the main polyphenols found in strawberries and apples. In strawberries, we analyzed F3OLs/PACs, ATs, ETs, and EA. Whereas for apple, F3OLs/PACs, FOLs, DHCs, and HCAs were reviewed. The major databases for the topic were used (Web of Science, ScienceDirect, SCOPUS, PubMed, and Google Scholar). The search terms used were: polyphenols or proanthocyanidins or anthocyanins or ellagic acid or flavonols or dihydrochalcones or hydroxycinnamic acids and thermal treatment or high-pressure processing or high hydrostatic pressure and strawberry or apple.

The search was focused on studies published over the last 10 years. Articles were included: (i) if the phenols were quantified by high-performance liquid chromatography (HPLC); (ii) when at least three studies were found for strawberry and apple identifying the effect of the related processing technology; (iii) when there was a control of fresh strawberry and apple or their unprocessed products. Articles were excluded: (i) when the analyzed sample was a mix of more than one fruit; (ii) when the HPP processing was combined with any thermal treatment higher than 50 °C; (iii) when the TT led to a concentration or reduction in the final product, e.g., jams.

For comparison purposes, the quantification of the different strawberry and apple polyphenols in the literature were converted to mg/100 g FW (fresh weight) to provide values closer to the fruit servings used in nutritional studies. The conversion of values based on DW (dry weight) to FW assumed that a fruit retains 10% of the original fresh weight after drying.

Overall, 26 articles were reviewed to address the study question: what are the effects of TT and HPP on the stability of polyphenols?

**Figure 2:**
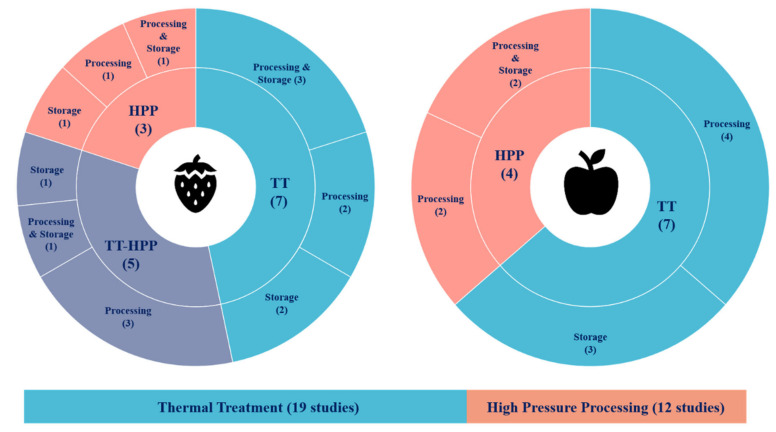
Overview of the studies evaluating the thermal treatment (TT) and high-pressure processing (HPP) impact and their subsequent storage on the primary polyphenols in strawberry and apple products.

- Strawberry was the most studied fruit with 15 articles, whereas 11 were examined for apple products;
- A total of 19 articles reported the effects of thermal treatment and 12 for high-pressure processing. The reviewed studies examined 199 different trials at different processing and storage conditions (Figure 2).

The concentrations reported in these studies for phenols of the same family were added. Each group’s total polyphenols were compared before and after processing, obtaining a percentage of increase or decrease attributed to the processing effect. A total of 4 of the 26 articles reviewed did not provide quantitative information to calculate these percentages, and therefore they were not included in the figures. The phenolic concentrations recorded after processing were considered control to determine the storage effect, and the results obtained during storage were compared against the control.

## 3. Effects of Processing and Storage Conditions on the Stability of Polyphenols

This section will discuss the positive and negative effects of TT and HPP treatments and their subsequent storage on the stability of the main polyphenols in strawberries (Table 1) and apples (Table 2). A summary is presented in Table 3.

### 3.1. Effects on the Stability of Proanthocyanidins in Strawberry and Apple Products

Proanthocyanidins, or condensed tannins, are plant secondary metabolites that could be found as oligomers or polymers of flavan-3-ol monomeric units. The most common monomeric building blocks are catechin, (epi)-catechin, (epi)-afzelechin, and (epi)-gallocatechin [28]. F3OLs/PACs are found in a wide variety of fruits, nuts, legumes, and cereals. The ranges reported highly depend on whether the study measured flavan-3-ol monomers (F3OLs), oligomers, and polymers or flavanol-3-ol units after hydrolysis (PACs). For most of the studies published, F3OLs and PACs were not differentiated, and therefore here we consider them together (F3OLs/PACs) unless differentiation is made.

In the case of strawberry, Nowicka et al. [3] recorded ranges of PACs polymers between 3.4 and 37.5 mg/100 g FW, while Buendia et al. [6] registered levels of total flavan-3-ol monomers from 53.9 to 168.1 mg/100 g FW after phloroglucinolysis. For apple, studies reported PACs polymers concentration from 12.6 to 203.3 mg/100 g FW in the whole fruit [12,29]. Nevertheless, higher levels were quantified for F3OLs units in peel and flesh. The flesh has from 1.4 to 548.2 mg/100 g FW, whereas in the peel, levels varied between 41.4 and 171.2 mg/100 g FW [7]. Numerous health benefits have been attributed to F3OLs/PACs, such as free radical scavenging, antioxidant, antiviral, antibacterial, anti-carcinogenic, anti-inflammatory, and anti-allergic activities [28,30].

For TT, a total of 8 studies with 30 trials including heat treatments from 70 to 98 °C during 0.4 to 15 min provided concentration data to calculate percentages of change of total F3OLs/PACs. Increments (10 trials) from 4% to 1800% were observed on total F3OLs/PACs in apple puree and juice, pasteurized strawberry and strawberry pulp, and puree [32,33,40,44,46,48]. Conversely, reductions (20 trials) from 16% to 75% were reported in strawberry puree, applesauce, and apple juice [7,33,46,50]. Whereas for HPP, a total of six studies with 28 trials under the following pressurization conditions, 300 to 600 MPa for 1 to 15 min at 22–35 °C were examined. Positive effects (13 trials) from no differences to 58% increments were observed in pressurized apples, apple juice, and strawberry puree [12,40,44,52,53]. However, decreases (15 trials) from 7% to 23% were reported in strawberry pulp, pressurized apples, and cloudy apple juice [12,44,54] (Table 3).

**Figure 3:**
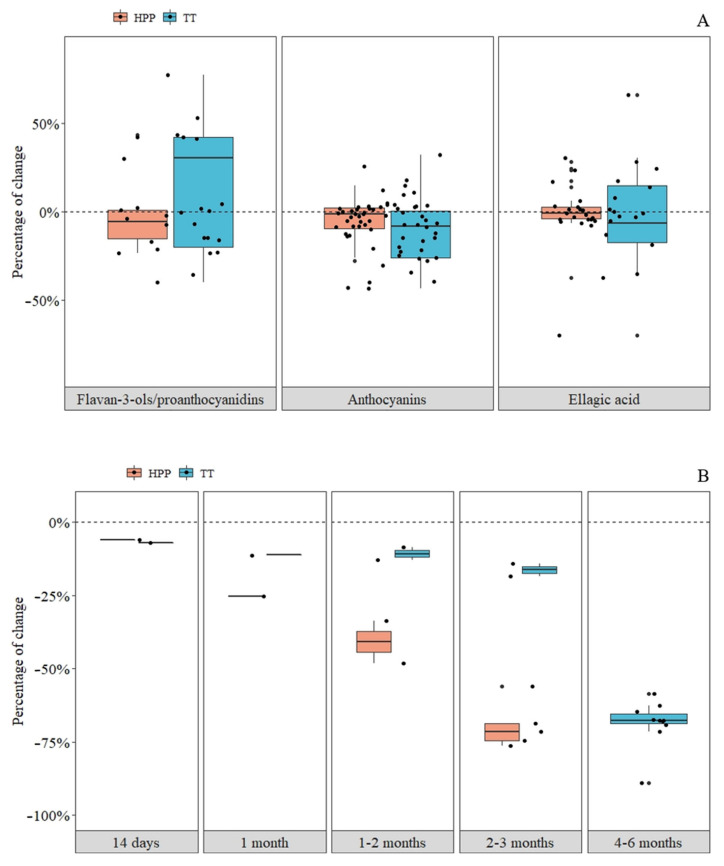
Impact of thermal treatment (TT) and high-pressure processing (HPP) on total flavan-3-ols/proanthocyanidins, anthocyanins, ellagic acid after different processing conditions (**A**), and on anthocyanins storage at 4–6 °C (**B**) in strawberry products. Each point represents the results of a trial. The percentages of change with the processing of each polyphenol family were calculated by adding the concentration of all polyphenols from the same family and comparing it before and after processing.

**Figure 4:**
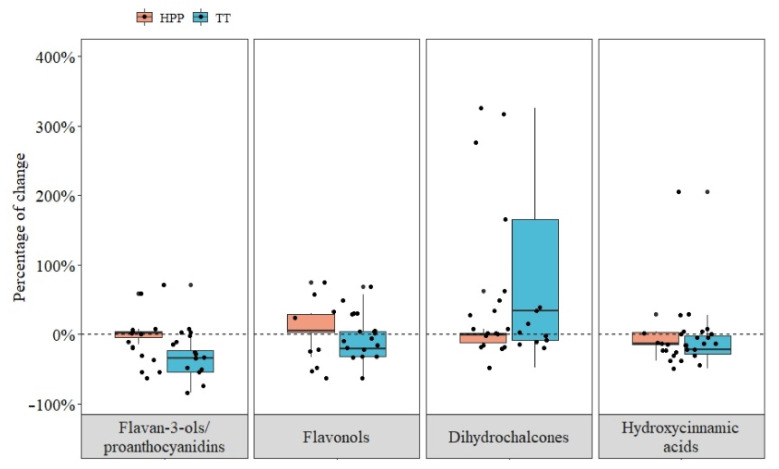
Impact of thermal treatment (TT) and high-pressure processing (HPP) on total flavan-3-ols/proanthocyanidins, flavonols, dihydrochalcones, and hydroxycinnamic acids after different processing conditions in apple products. Each point represents the results of a trial. The percentages of change with the processing of each polyphenol family were calculated by adding the concentration of all polyphenols from the same family and comparing it before and after processing. In TT, one trial with a percentage of change of F3OLs/PACs of 1800%, another with a percentage of change in DHCs of 768%, and another one with a percentage of change of HCAs of 925% were removed from the figures for scale reasons.

Overall, HPP maintained the content of F3OLs/PACs closer to the fresh control in both fruits. In contrast, the impact of TT on the degree of change of F3OLs/PACs was positive or negative depending on the study, influenced by the matrix, the TT conditions, and the response of the fruit cultivar to the processing technology [6,7] (Figure 3A and Figure 4). In addition, it is important to keep in mind that the type of extraction highly influences the reported F3OLs/PACs concentrations and the analytical method used.

For TT, Stübler et al. [40] found a 122% increase in catechin and 33% in proanthocyanidin B1 levels after processing strawberry puree at 72 °C for 1 min [40]. An increase in catechin (42%) was also observed in strawberry pulp treated at 70 °C for 2 min [44]. In the same direction, Oliveira et al. [31,32] reported increments on (+)-catechin, (–)-epigallocatechin, (–)-epicatechin, and (–)-epigallocatechin gallate levels in entire strawberries pasteurized at 90 °C for 5 min. However, after 360 days of storage at –20 °C and 90 days at 23 °C, the concentration of the monomers decreased compared to the content evaluated immediately after processing, although they were still higher than the fresh control [31,32]. Conversely, a general increase in polymeric PACs was observed in thermally treated (90 °C/2 min) cloudy strawberry juice kept at 4 and 20 °C for six months [37]. In this study, the PACs increment during storage could be explained by the protective effect exerted by the food matrix since cloudy juice contains pectin, which formed colloidal suspensions that limit their degradation. Consistent with the positive impact of TT on strawberry products, Alongi et al. [48] recorded a 71% increase in total flavan-3-ols after mild pasteurization (71.7 °C/0.4 min) of apple juice and an 1800% increase after intense pasteurization (90 °C/14.8 min). This vast increase was not represented in Figure 4 for reasons of scale. An increase in flavan-3-ol monomers together with a decrease in seven PAC oligomers was also reported in cloudy apple juice treated at 80–145 °C for 120 min [51]. The positive effects observed in flavan-3-ols were attributed to the heat treatment, which could have promoted the increase in these flavan-3-ol monomers, aiding in the release from the cellular tissue or favoring the cleavage of complex PACs molecules into their structural monomers [32,40,48,51]. On the contrary, other studies have reported reductions in F3OLs/PACs, mainly monomers, in strawberry puree after treatment at 85 °C/3 min [33], in clear apple juice treated 20 min at different temperatures (25–75 °C) [47] and in apple juice after pre-pasteurization and pasteurization process (98 °C/3sec) [50]. A wide degradation range (20–75%) of total F3OLs/PACs was also observed in pasteurized applesauce from 12 varieties, showing the influence of the variety in the thermal treatment effects [7]. PACs degradation might be explained either by oxidation reactions due to insufficient inactivation of enzymes or because the monomers released into the food matrix are more prone to non-enzymatic reactions after processing and storage [32]. Kim et al. [46] proposed that not only heating (90 °C for 30 min) but also the presence of oxygen during processing affected the decreases observed for flavan-3-ols in apple puree.

As mentioned before, in general, HPP showed minor variation in F3OLs/PACs levels compared to control. In the case of strawberry puree pressurized at 600 MPa for 1 min, Stübler et al. [40] detected a rise of 19% on procyanidin B1 and 68% on catechin, which could be the result of the release of phenols to the food matrix from the disrupted cell walls. Similarly, Szczepańska et al. [52] registered about 8% increments in procyanidin B2 after 300 and 450 MPa, and 18% in apple juices subjected to 600 MPa and multi-pulsed pressurization (300 MPa x 3 pulses). An explanation for these results could be that higher pressurization induced a better extraction of procyanidin B2 from the tissue. However, catechin and epicatechin concentration decreased after all HPP treatments, and after 12 weeks of storage at 4 °C, procyanidin B2 and catechin were not detected in all the pressurized juices [52]. Similarly, Cao et al. [44] reported lower catechin concentrations (4–23%) after HPP (at 400, 500, and 600 MPa for 5, 10, 15, 20, and 25 min) than in the fresh strawberry pulp. Another study also found significant decreases of 13% and 45% on epicatechin and procyanidin B in cloudy apple juice after HPP (600 MPa for 5 min at 25 °C). However, no significant differences were reported on catechin [54]. Further degradations were reported for catechin, epicatechin, and procyanidin B1 after 12 weeks of storage at 4 °C. The highest degradation was recorded for procyanidin B1 (92%), which was not detected after week 4. In general, the degradation of flavan-3-ol monomers, oligomers, and polymers after pressurization and subsequent storage might be attributed to insufficient inactivation of PPO and POD, which led to oxidation reactions and brown polymer formation. As occurred with the TT, apple origin and variety showed a high impact on the processing effect by HPP. Fernández-Jalao et al. [12] reported a different behavior after pressurization in Spanish and Italian apples. In Spanish apples, 400 MPa showed increases of 4% in procyanidin B2, whereas 500 and 600 MPa resulted in degradation of all F3OLs/PACs. In apples from Italy, pressurization at 600 MPa, resulted in increments of 30% in catechin, 39% in procyanidin B2, 45% in epicatechin, 70% in trimers, and 240% in epicatechin dimers.

### 3.2. Effects on the Stability of Anthocyanins in Strawberry Products

Anthocyanins (ATs) are water-soluble compounds found in different tissues of the plant, such as leaves, roots, flowers, and fruits [55]. These play an essential role in the sensory attributes of food products, as they are responsible for the characteristic red, purple, and blue coloration in fruits [56]. Anthocyanins can be found as aglycones or glycosylated derivatives. The six most frequently found in foods are pelargonidin, cyanidin, malvidin, delphinidin, petunidin, and peonidin [57]. The highest AT levels are found in berries, currants, grapes, and tropical fruits [58]. In the case of fresh strawberries, AT concentrations from 3.7 to 64.9 mg/100 g FW have been reported [3,6]. Numerous in vitro and in vivo studies have recognized the potential effect on preventing neurodegenerative and cardiovascular diseases and antioxidant, anti-inflammatory, anti-obesity, anti-diabetic, and chemopreventive properties [59,60,61].

For TT, 7 studies with 23 trials comprising temperatures from 70 to 100 °C for 1 to 15 min provided concentration data to calculate percentages of change of total ATs. Reductions (18 trials) from 5% to 44% were observed in pasteurized strawberry, strawberry purees and pulp [32,33,36,41,42,44]. On the contrary, other authors reported increments (5 trials) from 2% to 32% in strawberry puree [33,40].

Regarding HPP, a total of 6 studies, including 40 trials with pressurization conditions from 100 to 600 MPa for 1 to 20 min at 0–50 °C, were analyzed. Degradations (24 trials) from 7% to 28% were reported in strawberry pulp and puree [38,39,41,44]. In contrast, positive effects (16 trials), from no differences to 15% increase, were recorded in the same products [38,40,44] (Table 3).

In general, just after pressurization, the levels of ATs were preserved close to those found in fresh strawberries. In contrast, a more significant impact was observed in the degree of change of ATs due to TT, with a downward trend in both cases (Figure 3A).

Since ATs are very chemically sensitive compounds, most of the studies registered the degradation of ATs after processing and storage. For instance, pasteurization of entire strawberries at 90 °C for 5 min resulted in a reduction of 5–35% of cyanidin-3-glucoside, pelargonidin-3-rutinoside and pelargonidin-3-glucoside [31,32]. Higher degradations of 87–92% of these ATs were observed during 90 days of storage at 23 °C [32]. Similar results with losses of around 90–93% were observed after storage for eight weeks at 25 °C of strawberry puree treated at 100 °C for 10 min [35]. In another study, the storage of strawberry puree (heated at 90 °C for 15 min) during 12 weeks at 6 °C led to a mean degradation of about 19% of these ATs [43]. Degradation of total ATs (22%) was also observed in the strawberry pulp after heating at 70 °C for 2 min [44] and in strawberry puree pasteurized 90 °C for 15 min (44% of reduction) [41]. In general, degradation of ATs can follow three possible processes; cleavage of covalent bonds, polymerization and derivatization [32,36,41,43]. Moreover, this decrease could be partially caused by condensation reactions of ATs with other phenolics to produce colored polymeric pigments, resulting in strawberry pulp browning.

Interestingly, many authors have reported different degradation percentages depending on the strawberry variety, concluding that the effect of TT and subsequent storage on ATs also depends on the response of the fruit variety to the treatment [36,37,42]. The stability of ATs is also highly influenced by the food matrix properties. High pH and ascorbic acid content in the food matrix accelerates the degradation rate of ATs [43,62,63,64].

Although overall TT led to degradation on ATs, some studies reported a positive impact after processing. Garzoli et al. [33] reported a slightly higher content (2–18%) on total ATs in pasteurized puree (85 °C/3 min) compared with fresh puree. In agreement, Stübler et al. [40] showed that heat-treated (72 °C for 1 min) strawberry puree incremented the individual ATs as follows: cyanidin-3-O-glucoside (40%), pelargonidin-3-O-glucoside (26%), pelargonidin-3-O-rutoside (22%), pelargonidin-3-O-malonyl-glucoside (34%), and pelargonidin-3-O-acetylglucoside (39%). These increments in ATs might be attributed to a higher extraction of ATs from the matrix due to the heat treatment [40]. As mentioned before, and can be observed in Figure 3A, the changes in ATs content after HPP were minor compared with TT. Some studies reported no significant changes after pressurization. For instance, Bodelón et al. [38] found no significant differences in ATs levels in strawberry puree after HPP at 100, 200, 300, and 400 MPa at 20 °C compared with the untreated puree. However, a slightly higher decrease in ATs was observed in the puree pressurized at 50 °C compared with the untreated control. In line with this, the levels of individual and total ATs in strawberry pulp showed no significant changes after HHP treatments, regardless of the applied pressures or treatment times [44]. These results further support the idea that ATs were stable after pressurization. Stübler et al. [40] reported minor non-significant increases (8–12%) on all the soluble individual ATs, in strawberry puree after pressurization at 600 MPa for 1 min at room temperature. One explanation for this apparent increase in ATs could be the release of these compounds from the intact cells to the surrounding matrix. In other works, slight but significant decreases of about 7% on total ATs were reported in strawberry puree after HPP at 300 and 500 MPa for 1, 5, and 15 min at 0 °C. Under the same pressurization conditions combined with 50 °C, there was a degradation of 14% on ATs [41]. In the samples pressurized without heat treatment, the oxidative enzyme activity (PPO and POD) did not change significantly, leading to oxidation reactions and thus ATs degradation. However, the higher degradation of ATs in the pressurized puree combined with heat treatment might be due to the formation of colorless chalcones and reduction in flavylium cations and quinoid bases as a consequence of the thermal treatment. In another study, significant losses of 15 and 21% of total ATs were recorded in strawberry puree from two-year crops treated with HPP at 300 and 600 MPa for 15 min at 50 °C [39]. In the same line, Terefe et al. [42] also reported losses of ATs after HPP at 600 MPa for 5 min at 20 °C at different percentages depending on the variety: Camarosa (22–28%), Ruby Gem (27–42%), and Festival varieties (17–20%). Similar behavior for all varieties was observed after three months of refrigerated storage with losses between 62% and 77%. Losses up to 69% were also observed when pressurized strawberry puree (500 MPa for 15 min at 50 °C) was stored for 12 weeks at 6 °C showing that even HPP in combination with temperature was not enough to inactivate enzymes and led to a significant degradation during storage [43].

The effect of refrigerated storage (4–6 °C), from 14 to 180 days, on total ATs after different TT and HPP conditions is presented in Figure 3B. Storage at 4–6 °C after TT was examined in three studies [37,42,43]. Two studies were discussed to analyze the effect of storage after HPP, but only one used HPP without heat treatment [42], and the other combined HPP with heat treatment of 50 °C [43]. The losses of ATs during TT and HPP storage were progressive with the time, observing, in general, higher losses in the storage of HPP products. Storage from three to six months after HPP was only reported in a study with clear and cloudy strawberry juices that were not included in the figure because samples were also subjected to a previous blanching process at 100 °C/1 min in order to inactive enzymes [45].

### 3.3. Effects on the Stability of Ellagic Acid in Strawberry Products

Ellagitannins (ETs) constitute complex molecules with variable water solubility structured by one or more hexahydroxydiphenoyl (HHDP) moieties, which could be hydrolyzed to release ellagic acid (EA) [65]. ETs and EA are predominantly found in pomegranates, berry fruits, oak-aged red wine, tropical fruits, and nuts. ETs concentration in strawberries ranges from 7.18 to 28.85 mg/100 g FW and EA between 0.9 and 14.8 mg/100 g FW [3,5] with a more significant contribution from achenes than from flesh [5,66]. The potential health benefits of ETs and EA are associated with the metabolites (urolithins) produced by the human gut microbiota [67,68]. The biological functions attributed to urolithins comprise anti-oxidant, neuroprotective, anti-microbial, anti-inflammatory, and anticancer properties [67,69,70]. Notably, there were no differences in urolithins’ production and urinary excretion between volunteers ingesting either fresh strawberries or thermally treated puree [71].

For TT, 5 studies with 10 trials including temperatures from 55 to 90 °C for 1 to 15 min provided concentration data to calculate percentages of change of EA. Increases (6 trials) from 8% to 66% were observed in pasteurized strawberries, strawberry pulp, and strawberry purees [32,40,41,44]. In contrast, only Álvarez-Fernández et al. [34] reported a slight EA degradation (4 trials) from 8% to 35% in strawberries. Whereas for HPP, 3 studies with 27 trials with pressurization conditions ranging from 100 to 600 MPa for 1 to 25 min at 0–50 °C were analyzed. Increments (13 trials) from non-differences to 28% were reported in strawberry puree [40,41], whereas 2 to 37% decreases (14 trials) were observed in strawberry pulp [44], showing that the increases observed were most probably due to the enhanced extraction from the achenes (Table 3).

In general, TT and HPP influenced the level of EA both positively and negatively depending on the conditions. Still, the most significant increases were observed as a result of heat treatment (Figure 3A).

For TT, Cao et al. [44] reported a 17% increment in EA content after heating strawberry pulp at 70 °C for 2 min. The rise of EA levels after processing could have resulted from the hydrolysis of ETs and the release from the cellular structures. In agreement, pasteurization of strawberry puree at 90 °C for 15 min led to an increase of 31% on EA [41], and only a limited increase of 8% was observed when a milder TT of 72 °C for 1 min was applied [40]. Likewise, pasteurized (90 °C/5 min) strawberries had 143% higher levels of EA when compared with fresh fruit. However, after 360 days of storage at −20 °C, EA decreased 65% compared to the control [31]. Another study showed a zero-order kinetic model for EA degradation, with a final reduction of 32% in pasteurized strawberries stored for 90 days at 23 °C. Degradation due to storage might be due to increased exposure of EA released from cell walls, leading to non-enzymatic oxidation reactions [32]. On the other hand, during storage of pasteurized strawberry pure (90 °C/15 min) at 6 °C for 12 weeks, EA levels raised until week 10 and then slightly decreased toward the end of storage [43]. An explanation for the increase in EA during storage might be attributed to progressive release from high molecular weight ETs present in the puree [43]. Although the general trend is to increase EA after TT, Álvarez-Fernández et al. [34] reported decreases in EA (8–35%) during the strawberry puree processing and in the final product in the 2011 and 2012 harvest.

For HPP, Cao et al. [44] reported significant losses from 2% to 37% on EA levels in strawberry puree after HPP at 400 MPa for 5, 10, 15, 20, and 25 min, and 500 MPa for 5, 10, and 15 min at room temperature. However, a minor and non-significant decrease of 3% EA was observed in strawberry puree pressurized at 600 MPa for 1 min at room temperature [40]. In agreement, Marszalek et al. [41] also reported that the levels of EA in fresh strawberry puree did not change significantly after HPP at 300 and 500 MPa for 1, 5, and 15 min at 0 °C. However, when combining the same pressurization conditions at 50 °C, a significant increase of 28% EA concentration was observed [41]. These increments could be due to a release of EA from ETs due to the combination of HPP with temperature [72]. An EA increase of 43% was reported when strawberry puree pressurized at 500 MPa for 15 min at 50 °C was stored for 12 weeks at 6 °C [43].

### 3.4. Effects on the Stability of Flavonols in Apple Products

Flavonols (FOLs) are plant secondary metabolites that could be found in foods as aglycones or much more frequently as glycosidic conjugates [73]. The most commonly found in foodstuff are quercetin, kaempferol, myricetin, and isorhamnetin [74]. Although these are present in various fruits and vegetables, the major sources of FOLs are capers, saffron, onion, and tea [74,75]. Among the FOLs, quercetin glycosides are the most frequently found in apples. These are located mainly in the peel in concentrations from 5.3 to 119.7 mg/100 g FW [4]. However, some studies also reported minor amounts in the flesh [9,13]. Numerous studies have reported the benefits of FOL consumption on preventing cardiovascular diseases, diabetes, inflammation, viral infections, neurodegeneration, and cancer [76,77,78].

For TT, 3 studies with 17 trials including temperatures from 71 to 98 °C during 0.4 to 15 min provided concentration data to calculate percentages of change of total FOLs. Increments ranging from 4% to 69% were observed in five trials with apple juice and applesauce [7,48]. In contrast, reductions (12 trials) from 32% to 63% were detected in other studies with apple juice and applesauce [7,50]. A total of 3 studies with 11 trials under the following pressurization conditions, 300 to 600 MPa for 5 to 15 min at 22–35 °C, were analyzed after HPP. Positive effects ranged between 1% and 75% in nine trials after pressurization in entire apples and apple juice [12,52,53]. Conversely, decreases (2 trials) from 16% to 33% were also observed in pressurized apples [12] (Table 3).

Overall, HPP had positive effects on FOLs concentration. With TT, the results depended on the conditions and the apple varieties with a general trend to decrease their content (Figure 4).

For instance, FOLs contents increased 49% and 69% after mild (71 °C/0.4 min) and intense (90 °C/14.8 min) pasteurization of apple juice, respectively, which indicates that thermal treatment favored the release of phenolic compounds and reduced PPO activity [48]. Additionally, the thermal treatment also increased the bioaccessibility of FOLs compared to the fresh sample [79]. On the contrary, pre-pasteurization (98 °C/30 sec) and pasteurization (98 °C/50 sec) of apple juice from Red Fuji variety reduced rutin (64%), hyperin (86%), and quercetin levels (55%). A possible explanation for this decline might be the incomplete inactivation of PPO and POD enzymes by milder heat treatments [50]. The occurrence of these enzymes should be determined after the TT conditions. In another study with applesauce (12 varieties), it was impossible to calculate exactly the change percentage of FOLs after TT. Concentration before processing was reported separately in fresh flesh and peel [7]. In this case, 95% of flesh and 5% of peel were considered to calculate the concentration in the fresh fruit (control sample). Like in apple peel, six quercetin glycosides were found in applesauce in this order of importance: quercetin-3-galactoside > quercetin-3-arabinopyranoside > quercetin-3-rhamnoside > quercetin-3-glucoside > quercetin-3-xyloside > quercetin-3-rutinoside. After crushing, cooking (95 °C/2 min), and pasteurization (90 °C/5 min), different behavior was observed depending on the variety: increments of FOLs from 4% to 57% were observed in three varieties, and decreases from 6% to 63% were observed in 9 varieties. FOLs in the applesauce could result from the diffusion of quercetin derivatives from the peel during crushing or from the presence of small particles of peel retained in the sauce. Differences in the percentages between varieties could be explained by differences in the skin of apples, different structures, mechanical resistance, and cuticle thickness.

About HPP, no significant differences were reported in quercetin levels after apple juice pressurization at 300 and 450 MPa for 5 min and in multi-pulsed HPP at 300 MPa x 3 pulses, each 5 min. However, after HPP treatment at 600 MPa for 5 min, a minor but significant increase from 0.73 to 0.91 mg/L (25%) was quantified for quercetin [52]. These results could be attributed to an enhanced extraction from the juice tissue due to the high pressurization. Quercetin was not detected after two weeks of storage at 4 °C in all the treatments, and this degradation might be attributed to residual enzyme activity, which led to oxidation reactions [52]. Increases (9–35%) in different glycoside conjugates of quercetin were also found between the untreated and pressurized apples at 400 MPa for 5 min [12]. As for other phenolic compounds, FOLs stability also depends on the fruit variety, degree of ripening, and food matrix characteristics (pH, sugar content, and presence/absence of oxygen), which influence the behavior of the enzymes [12,53]. In this sense, Fernández-Jalao et al. [12] concluded that individual and total FOLs levels after pressurization were affected by HPP conditions and the apple origin. In terms of total FOLs concentration, the best results were obtained after pressurization at 400 MPa in Spanish apples, increasing 30%. However in 500 and 600 MPa led to significant reductions of quercetin-3-rutinoside (40–50%), quercetin-3-galactoside (33–53%) and quercetin-3-glucoside (24–46%) [12]. The degradation of quercetin glycosides might result from oxidation reactions caused by the residual activity of PPO and POD. In Italian apples, all the pressurization treatments increased quercetin glycosides, but the highest increase of about 75% was reported for HPP at 600 MPa [12]. The increase in individual and total FOLs content after HPP might be attributed to a change in the cell walls permeability and/or by disruption of the cell membranes, promoting a better extractability from cellular tissues [53].

### 3.5. Effects on the Stability of Dihydrochalcones in Apple Products

The dihydrochalcones (DHCs), phloretin and phloridzin, are characteristic compounds of apple and apple products. Since these compounds are exclusive of apples, these have been used to detect adulterations [80]. DHCs levels in entire apples ranged from 0.011 to 0.043 mg/100 g FW [11,12]. Although the DHCs could be found in flesh and peel, the highest concentration is located in the seeds, ranging from 24.1 to 86.4 mg/100 g FW [8]. Numerous studies have shown that both phloridzin and phloretin exert antibacterial, anti-inflammatory, antihyperglycemic, anti-diabetic and anticancer activities, and cardioprotective, neuroprotective hepatoprotective, and immunomodulatory properties [81,82,83].

For TT, 3 studies with 16 trials comprising thermal treatments from 71 to 98 °C during 0.4 to 15 min provided concentration data to calculate percentages of change of total DHCs. Increments (12 trials) ranged from 8% to 767% in applesauce and apple juice [7,48]. Conversely, reductions (4 trials) from 8% to 48% in apple juice and applesauce were recorded [7,50]. For HPP, four studies with 12 trials at pressurization conditions from 300 to 600 MPa for 5 to 15 min at 22–35 °C were analyzed. Positive effects (6 trials) showing from no differences to 63% increase were noted in pressurized apples and apple juice [12,52]. Conversely, reductions (6 trials) from 2% to 19% were reported for pressurized apples and cloudy apple juice [12,53,54] (Table 3).

Overall, TT and HPP changed the content of DHCs of apple products (Figure 4) positively. These differences are mainly explained by the use of whole apples during crushing in industrial processing, which contributes to releasing these compounds from the peel and seeds [84].

For TT, Le Bourvellec et al. [7] reported high variability on change percentages of DHCs in applesauce depending on the variety. Increments up to 325% were observed in nine varieties after crushing, cooking (95 °C/2 min) and pasteurization (90 °C/5) min compared with fresh control (calculated as 95% of flesh + 5% of peel). An explanation for the higher DHCs levels after processing could be the little peel and seed particles in the applesauce [7,85]. One interesting finding in this study was identifying a colorless phloridzin oxidation product, which indicated limited enzymatic oxidation due to processing. In line with this, Alongi et al. [48] detected increases of 165% and 767% on total DHCs in apple juice after mild (71 °C/0.4 min) and intense (90 °C/14.8 min) pasteurizations, respectively. These results confirm the association that crushing and heat treatment increase the release of phenols from peel and seeds. In contrast, Tian et al. [50] reported 18% and 48% reduction in phloridzin levels after pre-pasteurization (98 °C/30 sec) and pasteurization (98 °C/30 sec) respectively, probably due to a thermal degradation.

For HPP, Szczepańska et al. [52] reported no significant differences in phloridzin concentration in apple juice pressurized at 300, 450, and 600 MPa, and multi-pulsed 300 MPa x 3 pulses for 5 min. However, after 12 weeks of refrigerated storage, phloridzin concentration was reduced between 71% and 84% depending on the HPP conditions. In another study, after HPP at 600 MPa for 5 min at 25 °C, phloridzin levels changed from 48.8 to 40.2 mg/L, representing losses of 18%. After two weeks of storage at 4 °C, 51% decreases were observed, reaching up to 71% losses at the end of 12 weeks of storage [54]. As for FOLs, the degradation in DHCs was mainly due to HPP conditions that were insufficient to inactivate enzymes (PPO and POD). Therefore, the phenolic compounds underwent oxidation reactions. Fernández-Jalao et al. [12] indicated that the apple origin conditioned the effects of HPP on DHCs. In Spanish apples, 500 and 600 MPa treatments led to reductions in phloridzin (16–20%) and phloretin-2′-xylosylglucoside (14–17%) levels. Conversely, in Italian apples, HPP at 600 MPa increased 51% and 67% the phloretin-2′-xylosylglucoside and phloridzin concentrations, respectively.

### 3.6. Effects on the Stability of Hydroxycinnamic Acids in Apple Products

Hydroxycinnamic acids (HCAs) are the major subgroup of phenolic acids and may occur either in their free or conjugated forms, including amides, esters, and glycosides [86]. The main aglycones identified in foodstuff are *p*-coumaric, caffeic, ferulic, and sinapic acids [87]. More frequently found in coffee, various fruits, some vegetables, and whole grains [88]. In apples, chlorogenic acid (5-O-caffeoylquinic) is the predominant compound. The HCA levels vary depending on the parts of the fruit. In apples, the flesh is the structure characterized for the higher levels of HCAs [89]. Ranges in whole apples were from 2.5 to 23.1 mg/100 g FW [11,12]. Whereas in apple flesh, HCAs levels varied from 0.7 to 14.3 mg/100 g FW [9,10]. Some potential health benefits have been documented from HCAs intakes, such as anti-microbial, anti-diabetic, antioxidant activity, prevention of cardiovascular and neurodegenerative diseases, and some cancer conditions [87,90,91].

For TT, four studies with 19 trials comprising heat treatments from 71 to 98 °C for 0.4 to 30 min provided concentrations data to calculate percentages of change of total HCAs. Increments (5 trials) ranged from 8% to 925% in apple sauce and apple juice [7,48]. On the other hand, reductions (14 trials) fluctuated from 4% to 49% in apple puree, sauce, and juice [7,46,50]. For HPP, four studies with 12 trials with pressurization conditions from 300 to 600 MPa for 5 to 15 min at 22 to 35 °C were reviewed. Positive effects (5 trials) from no significant differences to increments up to 29% were recorded in entire apples and apple juice [12,52]. Conversely, losses (7 trials) varied from 12% to 39% in whole apples and cloudy apple juice [10,53,54] (Table 3).

In general, both TT and HPP caused degradation of HCAs after processing. Nevertheless, although a higher degree of degradation was observed after heat treatment, it also caused the most significant increase in two trials with apple juice (the highest one is not shown in the graph for scale reasons) (Figure 4).

For TT, Alongi et al. [48] identified the highest increases in HCAs after heat treatment, observed in both chlorogenic acid and *p*-coumaroylquinic acid. A total of 205% and 925% increments on total HCAs were reported after the mild (71 °C/0.4 min) and intense (90 °C/15 min) pasteurization of apple juice, respectively. The increase in HCAs could result from the favoring effect of thermal treatment in enzyme inactivation and the release of chlorogenic acid from the cell walls to the food matrix [7,48]. These results corroborate the finding of De Paepe et al. [51], who concluded that phloretin-2´-O-glucoside and 3-O-caffeoylquinic acid were thermal-resistant compounds in cloudy apple juice isothermally treated from 80 to 145 °C during 7200 s. In contrast, Tian et al. [50] reported a 15% and 30% degradation of chlorogenic acid levels in apple juice after pre-pasteurization (98 °C/30 sec) and pasteurization (98 °C/50 sec) respectively. In agreement with these results, a significant decrease of 44% in chlorogenic acid was recorded in apple puree heated at 90 °C for 30 min in the presence of oxygen after storage. However, no effect was observed in the heat-treated puree in the absence of oxygen [46], suggesting that oxidation reactions during heating are the main reason for the HCAs degradation [46,50]. In addition, Le Bourvellec et al. [7] reported that the higher differences on 5´-caffeoylquinic acid and *p*-coumaroylquinic acid after TT were due to apple variety. After cooking (95 °C/2 min) and pasteurization (90 °C/5 min), nine varieties experienced reductions from 1% to 48% in 5’-caffeoylquinic acid, whereas increases of 8%, 4%, and 30% were observed in Golden Delicious, Freiberg, and Granny Smith varieties, respectively.

For HPP, Marszalek et al. [54] found no significant differences in chlorogenic acid concentration of cloudy apple juice due to pressurization at 600 MPa for 5 min at 25 °C. However, there was a progressive degradation up to 53% after 12 weeks of storage at 4 °C. Accordingly, Szczepańska et al. [52] reported no differences in chlorogenic acid levels after static HPP at 300 MPa for 5 min and multi-pulsed pressurization (300 MPa × 3 pulses). However, a slight increase of around 5% was observed on the pressurized juices at 450 and 600 MPa for 5 min. Refrigerated storage for 12 weeks produced substantial losses of chlorogenic acid (66–77%). Reductions of 12% for total HCAs were reported for apples pressurized at 400 MPa for 5 min [53]. In another study, Fernández-Jalao et al. [12] reported that the HPP effect on individual and total HCAs was associated with the apple origin. In Spanish apples, pressurization reduced all individual HCAs, neochlorogenic, cryptochlorogenic, coumaroylquinic, and chlorogenic acids. The latter is the one that suffered the most significant degradations, with losses of 44% at 400 MPa, 24% at 500 MPa, and 15% at 600 MPa. Whereas in Italian apples, HPP at 600 MPa increased all the individual HCAs, resulting in a total increment of 29%. In contrast, HPP at 400 and 500 MPa reduced total HCAs by 16% and 13%, respectively.

## 4. Conclusions

This review analyzed the effects of TT and HPP treatments on the phenolic compounds in strawberry (F3OLs/PACs, ATs, and EA) and apple (F3OLs/PACs, FOLs, DHCs, and HCAs) products. Our findings show that the effect on polyphenols content (positive or negative) was contingent upon the type of processing, type of fruit, polyphenol family, and the shelf-life conditions (time and temperature during storage) of the final product. The impact of TT relied mainly on the food matrix and the thermal stability of the different phenolic compounds.

TT had positive effects in strawberry products, as was observed for F3OLs/PACs and EA. However, it had negative effects on the ATs content. These were due to the thermal instability of ATs and the enhanced extraction of EA and condensed tannins from achenes with thermal treatments. It is well known that ATs are heat-labile compounds, susceptible to oxidation and condensation reactions, which was confirmed in most of the studies where degradation of ATs was observed after TT. On the contrary, TT increased EA content due to the hydrolysis from ETs and release from the lignified matrix of the achenes.

In apple products, TT had positive effects, significantly promoting the release of FOLs and DHCs from peel and seeds. However, most of the studies observed a negative effect of F·OL/PACs, principally due to the extraction processes that quantified flavan-3-ols oligomers rather than monomers. Nevertheless, the final concentrations were variable depending on the variety, and the TT conditions applied. HPP treatments maintained the concentrations of phenolic compounds closer to those of the fresh or unprocessed samples regardless of the food matrix.

The impact of storage after TT and HPP had only been described for ATs in strawberry products. In general, TT has positive effects preserving better the ATs during storage. However, in the only study that examined the effect of HPP on ATs during storage, a negative effect was described showing a higher degradation than TT. Negative effects were also observed as the fast and pronounced degradation of FOLs, DHCs, and HCAs in apple products after HPP and storage.

The phenolic compounds’ degradation after storage of HPP products could be due to the limitations in oxidative enzyme inactivation when thermal treatments were insufficient. From the industrial perspective, manufacturers aiming to preserve the natural content of polyphenols need to find the sweet spot between polyphenol stability and product shelf life. Further studies are recommended to compare how both technologies influence the content of the different families of polyphenols and their bioavailability and bioactivity.

## Figures and Tables

**Table 1:** Effects of TT and HPP treatments and storage on the main phenolic compounds in strawberry products.

Material	Treatment: Conditions	Storage	Impact of Processing Conditions on Polyphenols ^a^	Impact of Storage Conditions on Polyphenols ^b^	Mechanisms	Ref.
Strawberry fresh	TT: 90 °C/5 min	360 days −20 °C	F3OLs/PACs: ↑30% CA, ↑73% EGC; ↑45% EC ATs: ↓16% pel-3-glu; ↓5% pel-3-rut; ≈cya-3-glu EA: ↑143%	F3OLs/PACs: ↓19% CA, ↓39% EGC ATs: ↓pel-3-glu; ↓pel-3-rut; ↑cya-3-glu EA: ↓65%	↑F3OLs/PACs: cleavage → release of dimers and monomers; release from cellular tissue ↓ATs: cleavage of covalent bonds, polymerization, and derivatization; ↑pH in the food matrix; enzymatic oxidation after pasteurization and during storage ↑EA: ETs hydrolysis	[31] ^d^
Strawberry fresh	TT: 90 °C/5 min	90 days 23 °C	F3OLs/PACs: ↑34% CA; ↑134% EC; ↑119% EGCG; *↑30% T.F3OLs/PACs* ATs: ↓30% cya-3-glu; ↓35% pel-3-glu; ≈ pel-3-rut; *↓30% T.ATs* EA: ↑66%	F3OLs/PACs: ↓42% CA; ↓62% EGCG; ↓67% EC ATs: ↓87% cya-3-glu, ↓97% pel-3-glu; ↓92%pel-3-rut EA: ↓32%	TT ↑F3OLs/PACs: PACs cleavage → release of dimers and monomers; release from cellular tissue ↓ATs: cleavage of covalent bonds, polymerization, and derivatization ↑EA: release from cell walls; hydrolysis from ETs to EA Storage ↓F3OLs/PACs, ↓ATs and ↓EA: oxidation; non-ezymatic and enzymatic oxidation	[32]
Strawberry puree	TT1 (SB): 85 °C/3 min TT2 (P): 85 °C/3 min	No	SB: F3OLs/PACs: from *↑42% to↓16%* ATs: *↓34–43%* P: F3OLs/PACs: from *↑53% to ↓35%* ATs: *↑2–18%*	-	↓F3OLs/PACs: TT conditions degraded heat-labile flavan-3-ols ↓ATs: SB may influence the ATs stability	[33]
Strawberry puree (2 yearsharvest )	TT: 90 °C/2 min	No	Puree with seeds EA: ↓8% (2011 harvest), ↓13% (2012 harvest) Puree without seeds EA: ↓35% (2011 harvest) ↓19% EA (2012 harvest)	-	↓EA: oxidation by membrane breakage	[34]
Strawberry puree	TT: 100 °C/10 min	8 weeks 25 °C	-	ATs: ↓80–88% pel-3-glu (8 weeks); ↓53–74% pel-3-rut (2 weeks); ↓63–78% pel-3-mal-glu (2 weeks); ↓70–86% pel-3-ace-glu (2 weeks); *↓90–93% T.ATs (8 weeks)*	↓ATs: oxidation by PPO; formation of dark condensation products	[35]
Strawberry puree (2 varieties)	TT1 (SB): 100 °C/3 min TT2 (P): 60, 75, 90 °C/3 min	28 days 20 °C	Ats—Elsanta var.: from *≈ to ↓5% (P60; P75); ↓10–12% (SB; P90)* ATs—Everest var.: *≈ (P90); ↓8% (SB, P60, P75)*	Elsanta var.: *↓62–65% T.ATs* (all treatments) Everest var.: *↓64–73% T.ATs* (all treatments)	SB and P ↓ATs: cleavage of covalent bonds, polymerization, and derivatization Storage ↓ATs: PPO partial reactivation during storage → oxidation	[36]
Strawberry juice (7 varieties)	TT: 90 °C/2 min	6 months at 4 and 20 °C	-	4 °C: F3OLs/PACs: from*↓0.3% to ↑27% polymeric PACs* ATs: ↓69% pel-3-glu; ↓73% pel-3-mal-lglu; ↓68% cya-3-glu; ↓56% cya-3-mal-glu; *↓59–89% T.ATs* 20 °C: F3OLs/PACs: from *↓10% to ↑11% polymeric PACs* ATs: ↓97% pel-3-glu; ↓99% pel-3-malonylglu; ↓98% cya-3-glu; ↓85% cya-3-mal-glu; *↓94–99% T.ATs*	↑F3OLs/PACs: protective effect of colloidal suspensions ↓F3OLs/PACs and ↓ATs: cleavage of covalent bonds, polymerization, and derivatization; enzymatic oxidation after pasteurization and during storage	[37]
Strawberry puree	HPP: 100–400 MPa /15 min at 20 and 50 °C	No	ATs: ≈ pel-3-glu; ≈cya-3-glu; ≈pel-3-rut (all HPP); from *≈ to ↑15% T.ATs*	-	≈ ATs: Sufficient enzyme inactivation	[38]
Strawberry puree (2 years harvest)	HPP: 300 and 600 MPa/ 15 min at 50 °C	28 weeks 6 °C	ATs - 300 MPa harvest 2011: ↓12% cy-3-glu; ↓13% pel-3-glu; ↓36% pel-3-rut; *↓15% T.ATs* ATs - 600 MPa harvest 2012: ↓22% cy-3-glu; ↓21% pel-3-glu; ↓10% pel-3-rut; *↓21% T.ATs*	ATs–300 MPa harvest 2011: 86 days half-life ATs–600 MPa harvest 2012: 62 days half-life	↓ATs: residual PPO activity → oxidation	[39]
Strawberry pure	TT: 72 °C/1 min HPP: 600 MPa/1 min	No	TT F3OLs/PACs: ↑122% CA; ↑33% proanthocyanidin B1; *↑78% T.F3OLs/PACs* ATs: ↑40% cya-3-O-glu; ↑26% pel-3-O-glu; ↑22% pel-3-O-rut; ↑34% pel-3-O-mal-glu; ↑39% pel-3-O-acetylglu; *↑32% T.ATs* EA: ↑8% HPP F3OLs/PACs: ↑68% CA; ↑19% proanthocyanidin B1; *↑43% T.F3OLs/PACs* ATs: ↑12% cya-3-O-glu; ↑8% pel-3-O-glu; ↑10% pel-3-O-rut; ↑13% pel-3-O-mal-glu; ↑12% pel-3-O-ace-glu; *↑11% T.ATs* EA: ≈	-	TT ↑F3OLs/PACs: cleavage → release of dimers and monomers ↑ATs: higher extraction from cell matrix favored by TT HPP: ↑F3OLs/PACs: release from the disrupted cell walls	[40]
Strawberry puree	TT: 90 °C for 15 min HPP: 300 and 500 MPa/ 1, 5, 15 min at 0 °C	No	TT ATs: ↓44% cya-3-glu; ↓43% pel-3-glu; ↓49% pel-3-rut; *↓44% T.ATs* EA: ↑30.5% EA HPP – 0 °C (all HPP conditions) ATs: ↓5% cya-3-glu; ↓7% pel-3-glu; ↓15% pel-3-rut; *↓7% T. ATs* EA: ≈ EA (300 and 600 MPa) HPP + 50 °C ATs: ↓14% cya-3-glu; ↓13% pel-3-glu; ↓31% pel-3-rut; *↓14% T.ATs* EA: ↑28.4% (300 MPa); ↑15.5% (600 MPa)	-	TT ↓ATs: cleavage of covalent bonds, polymerization, and derivatization ↑EA: release from the achenes favored by TT HPP – 0 °C ↓ATs: insufficient enzyme inactivation (PPO and POD) → oxidation HPP + 50 °C ↓ATs: formation of colorless chalcones ↑EA: release from ETs	[41]
Strawberry Puree ^c^ (3 varieties)	TT: 88 °C/2 min HPP: 600 MPa/5 min at 20 °C	3 months 4 °C	TT Camarosa var. ATs: ↓16% cya-3-glu; ↓23% pel-3-glu; ↓26% pel-3-rut; *↓22% T.ATs* Rubygem var. ATs: ↓42% cya-3-glu; ↓24% pel-3-glu; ↓29% pel-3-rut; *↓25% T.ATs* Festival ATs: ↓27% cya-3-glu; ↓26% pel-3-glu; ↓26% pel-3-rut; *↓26% T.ATs* HPP Camarosa var. ATs: ↓22% cya-3-glu; ↓26% pel-3-glu; ↓28% pel-3-rut; *↓26% T.ATs* Rubygem var. ATs: ↓42% cya-3-glu; ↓27% pel-3-glu; ↓32% pel-3-rut; *↓28% T.ATs* Festival ATs: ↓17% cya-3-glu; ↓20% pel-3-glu; ↓18% pel-3-rut; *↓20% T.ATs*	TT Camarosa var. ATs: ↓66% cya-3-glu; ↓69% pel-3-glu; ↓60% pel-3-rut; *↓68% T.ATs* Rubygem var. ATs: ↓52% cya-3-glu; ↓69% pel-3-glu; ↓59% pel-3-rut; *↓68% T.ATs* Festival ATs: ↓59% cya-3-glu; ↓65% pel-3-glu; ↓59% pel-3-rut; *↓65% T.ATs* HPP Camarosa var. ATs: ↓69% cya-3-glu; ↓72% pel-3-glu; ↓69% pel-3-rut; *↓72% T.ATs* Rubygem var. ATs: ↓62% cya-3-glu; ↓75% pel-3-glu; ↓71% pel-3-rut; *↓75% T.ATs* Festival ATs: ↓73% cya-3-glu; ↓77% pel-3-glu; ↓76% pel-3-rut; *↓76% T.ATs*	↓ATs: partially due to variety effect PPO and POD; oxidation and co-oxidation; non-enzymatic reactions; cleavage of covalent bonds	[42]
Strawberry pure	TT: 90 °C for 15 min HPP: 500 MPa/15 min at 50 °C	12 weeks 6 °C	-	TT ATs: ↓17% cya-3-glu; ↓19% pel-3-glu; ↓19% pel-3-rut; *↓19% T.ATs* EA: ↑ 56% EA until week 10 HPP+50 °C ATs: ↓72% cya-3-glu; ↓68% pel-3-glu; ↓72% pel-3-rut; *↓69% T.ATs* EA: ↑43% EA until the end of storage	TT-Storage ↑EA: release from the achenes; ETs hydrolysis; low pH increased ETs hydrolysis HPP Storage ↓ATs: not enough enzyme inactivation PPO and POD → oxidation	[43]
Strawberry pulp	TT: 70 °C/2 min HPP: 400, 500, 600 MPa/ 5, 10, 15, 20, 25 min at 25 °C	No	TT F3OLs/PACs: ↑42% CA ATs: ↓17% cy-3-glu, ↓23% pel-3-glu, ↓21% pel-3-rut; *↓22% T.ATs* EA: ↑17% HPP F3OLs/PACs: ≈CA (500 MPa/20, 25 min; 600 MPa/5–25 min); ↓7–23% CA (400 MPa/5–25 min; 500 MPa/5–15 min); from *≈ to ↓23% T.F3OLs/PACs* ATs: ≈cy-3-glu, ≈pel-3-glu, ≈pel-3-rut, *≈T.ATs (all HPP conditions)* EA: ↓2–37% (400MPa/20 min; 500 MPa/5, 20 min; 600 MPa/10, 20, 25 min)	-	TT ↑F3OLs/PACs: extraction from the achenes favored by TT ↓ATs: condensation reactions with other phenols → browning; PPO and POD oxidation ↑EA: release from the achenes; ETs hydrolysis HPP ↓F3OLs/PACs: no complete enzyme inactivation (PPO and POD) → oxidation	[44]
Clear and cloud strawberry juices	TT (SB): 100 °C/1 min HPP: 600 MPa/4 min at 43 °C	6 months at 4 and 25 °C	-	Clear juice at 4 °C ATs: ↓10% cy-3-glu, ↓6% pel-3-glu, ↓9% pel-3-rut; *↓7% T.ATs* Cloudy juice at 4 °CATs: ↓26% cy-3-glu, ↓33% pel-3-glu, ↓21% pel-3-rut; *↓30% T.ATs* Clear and cloudy juice at 25 °C: T.ATs: *↓> 95%*	↓ATs: PPO and POD oxidation; condensation with other phenols → colorless compounds; oxidative degradation of ascorbic acid (especially at higher storage temperature)	[45]

↑: increment vrs. control; ↓: diminution vrs. control; ≈: unchanged vrs. control; >: higher than; TT: thermal treatment; SB: steam blanching; P: Pasteurization;HPP: high-pressure processing; F3OLs/PACs: flavan-3-ols/proanthocyanidins; ATs: anthocyanins; EA: ellagic acid; T.: total; CA: catechin; EC: epicatechin: ECG: epicatechin gallate; EGC: epigallocatechin; EGCG: epigallocatechin gallate; pel: pelargonidin; cya: cyanidin; glu: glucoside; rut: rutinoside; mal-glu: malonylglucoside; ace-glu: acetylglucoside; pen: pentoside; ara: arabinoside; PPO: polyphenol oxidase; POD: peroxidase; ^a^ Total percentage of change (in italic) was calculated by adding the concentration of all polyphenols from the same family and comparing it before and after processing. These data are represented in Figure 3 and Figure 4. Control samples were the same matrixes (juices, puree, etc.) just before TT or HPPP; ^b^ To study storage effects, samples after TT or HPPP at time 0 were used as control; ^c^ Control sample was the fresh fruit; ^d^ Studies without concentration data to calculate the percentage of change (kinetics or graphs).

**Table 2:** Effects of different TT and HPP treatments and storage on the main phenolic compounds in apple products.

Material	Treatment: Conditions	Storage	Impact of Processing Conditions on Polyphenols ^a^	Impact of Storage Conditions on Polyphenols ^b^	Mechanisms	Ref.
Apple pureec	TTwith O_2_: 90 °C/30 min + O2 TT ∅ O_2_: 90 °C/30 min ∅ O_2_	No	TTwith O_2_ F3OLs/PACs: ≈ EC; ≈procyanidin-dimer 1; ↓45% proanthocyanidin trimer; ↓62% CA; ↓30% T.F3OLs/PACs HCAs: ↓44% chlorogenic acid TT ∅ O_2_ F3OLs/PACs: ≈EC, ↑35% procyanidin-dimer 1, ≈proanthocyanidin trimer; ≈CA; ↑7% T.F3OLs/PACs HCAs: ≈chlorogenic acid	-	↓F3OLs/PACs and HCAs: oxidation reactions during heating	[46]
Clear apple juice	TT: 25, 35, 45, 55, 65 and 75 °C/20 min	No	F3OLs/PACs: ↓EC and CA HCAs: ↓chlorogenic acid	-	↓F3OLs/PACs and HCAs: enzymatic oxidation	[47]^d^
Apple juice	TT1: 71.7 °C/0.4 min TT2: 90 °C/14.8 min	No	TT1 F3OLs/PACs: *↑71% T.F3OLs/PACs* HCAs: ↑244% chlorogenic acid; ↑156% p-coumaoylquinic acid; *↑205% T.HCAs* DHCs: ↑156% phloretin xyloglucoside; ↑192% phloridzin; *↑165% T.DHCs* FOLs: ↑39% que-3-O-gal; ↑988% que-3-O-hex; ↑50% que-3-O-xyl; ↑7% que-3-O-rha; ↑33% que-3-O-pen; *↑49% T.FOLs* TT2 F3OLs/PACs: *↑1800% T.F3OLs/PACs* HCAs: ↑1352% chlorogenic acid; ↑389% p-coumaoylquinic acid; *↑925% T.HCAs* DHCs: ↑752% phloretin xyloglucoside; ↑808% phloridzin; *↑767% total DHCs* FOLs: ↑67% que-3-O-gal; ↑1113% que-3-O-hex; ↑92% que-3-O-xyl; ↑14% que-3-O-rha; ↑48% que-3-O-pen; *↑69% T.FOLs*	-	↑F3OLs/PACs: cleavage → release of dimers and monomers ↑FOLs: release from cells walls; ↓ PPO activity ↑DHCs: enhanced release from peel and seeds ↑HCAs: release from cells walls favored by TT; enzyme inactivation	[48]
Apple juice (2 years of harvest)	TT: 85 °C	360 days 4, 20, and 37 °C	-	4 and 20 °C: ≈ ∑ flavanols, DHCs, FOLs, and phenol carboxylic acids 37 °C: ↓ ∑ flavanols, DHCs, FOLs, and phenol carboxylic acids	↓ ∑ flavanols, DHCs, FOLs, and phenol carboxylic acids: Higly influenced by storage temperature	[49]^d^
Apple juice	TT1: 98 °C/30 sec TT2: 98 °C/30 sec	No	TT1 F3OLs/PACs: ↓32% CA; ↓31% EC; ↓33% EGC; ↓43% ECG; ↓18% procyanidin B2; *↓18% T.F3OLs/PACs* FOLs: ↓25% rutin; ↓50% hyperin; ↓27% que; *↓32% T.FOLs* DHCs: ↓ 18% phloridzin HCAs: ↓16% chlorogenic acid TT1+TT2 F3OLs/PACs: ↓58% CA; ↓56% EC; ↓59% EGC; ↓70% ECG; ↓37% procyanidin B2; *↓48% T.F3OLs/PACs* FOLs: ↓64% rutin; ↓86% hyperin; ↓55% que; *↓63% T.FOLs* DHCs: ↓ 48% phloridzin HCAs: ↓30% chlorogenic acid	-	↓F3OLs/PACs: TT conditions degraded heat-labile flavan-3-ols ↓FOLs: due to the discard of peel solids from the juice; the remaining enzyme activity ↓DHCs: due to thermal degradation	[50]
Apple sauce ^c^ (12 varieties)	TT1: 95 °C/2 min TT2: 95 °C/5 min	No	F3OLs/PACs: ↓20–85% procyanidins oligomers; ↓22–59% CA; ↓13–74% EC; *↓20–75% T.F3OLs/PACs* FOLs: from ↑*6–63% to* ↓*4–57% T.FOLs;* DHCs: from ↓50% to ↑54% phloridzin; ↑1–1285% phloretin-2-xyloglucoside; from ↓8–14% to ↑%*8–325% T.DHCs* HCAs: ↓1–47% 5’-caffeoylquinic acid (9 varieties); ↑4–30% 5’-caffeoylquinic acid (4 varieties); from *↓4–49% to ↑7–27% T. HCAs*		↑ and ↓ of phenols highly related to the apple variety ↓F3OLs/PACs: due to oxidation ↑ FOLs: diffusion of quercetin glycosides from the peel to the applesauce ↑DHCs: diffusion from the seeds to the applesauce ↑HCAs: release from cells walls favored by TT; Enzyme inactivation	[7]
Cloudy apple juice^c^	TT: 80–145 °C/over 7200 sec	No	F3OLs/PACs: ↓7 procyanidin oligomers (mainly B type); ↑ CA; ↑EC; ↑dimeric compounds	-	↓F3OLs/PACs oligomers: cleavage → release of dimers and monomers ↓FOLs: glycosidic bond hydrolisis in an acidic matrix	[51]^d^
Apple juice	HPP: 300, 300 (x3), 450, 600 MPa/5 min at 20 °C	12 weeks 4 °C	F3OLs/PACs: ≈CA (all HPP); ↓EC (all HPP); ↑8% procyanidin B2 (300 and 400 MPa); ↑18% procyanidin B2 (300 x3 and 600 MPa); from *≈ to ↑8% T. F3OLs/PACs* DHCs: ≈ phloridzin (300, 300x3, 450 MPa; 600 MPa) FOLs: ≈ que (300, 300x3, 450 MPa); ↑25% que (600 MPa); *↑1–25% T. FOLss* HCAs: ≈chlorogenic acid (300 and 300x3 MPa); ↑5% chlorogenic acid (450 and 600 MPa)	F3OLs/PACs: ∅ C and procyanidin B2 after 6 weeks (all HPP); ↓77% EC (except in 300 MPa) FOLs: ∅ quercetin after 2 weeks (all HPP) DHCs: ↓71–84% phloridzin (all HPP) HCAs: ↓66–77% chlorogenic acid (300x3, 450, 600 MPa)	HPP: higher pressurization → higher extraction from apple tissue ↑FOLs: release from the disrupted cell walls Storage ↓F3OLs/PACs and FOLs: oxidation reactions	[52]
Apples (Spain and Italy)	HPP: 400, 500, 600 MPa/5 min at 35 °C	No	Spanish apples: *400 MPa (best treatment)* F3OLs/PACs: ≈CA; ≈EC; ≈dimers; ≈trimers; ↑4% procyanidin B2; *≈ T.F3OLs/PACs* FOLs: ≈que-3-rut; ↑35% que-3-gal; ↑22% que-3-glu; ↑30% que-3-ara; ↑32% que-3-xyl; ↑33% que-3-rha; *↑30% T.FOLs* DHCs: ≈phloridzin; ↓9% phloretin-2-xyloglucoside; *↓2% T.DHCs* HCAs: ↓44% chlorogenic acid; ↓9% neochlorogenic acid; ↓10% cryptochlorogenic acid; ↓17% coumaroyl quinic acid; *↓39% T. HCAs* *500–600 MPa* F3OLs/PACs: ≈CA; ↓8–13% procyanidin B2; ≈trimers; ≈dimers; ↓11–17% EC; *↓11% T.F3OLs/PACs* FOLs:↓40–50% que-3-rut; ↓33–53% que-3-gal; ↓24–46% que-3-glu; ↓3–16% que-3-ara; ↓6–23% que-3-xyl; ↓15% que-3-rha; *↓16–33% T.FOLs* DHCs: ↓16–20% phloridzin; ↓14–17% phloretin-2-xyloglucoside; *↓15–19% T.DHCs* HCAs: ↓15–24% chlorogenic acid; ↓4% neochlorogenic acid; ↓12–14% cryptochlorogenic acid; ↓12–19% coumaroyl quinic acid; *↓14–22% T. HCAs* Italian apples: *600 MPa (best treatment)* F3OLs/PACs: ↑30% CA; ↑39% procyanidin B2; ↑70% trimers; ↑242% dimers; ↑45% EC; *↑58% T.F3OLs/PACs* FOLs: ↑ 88% que-3-rut; ↑107% que-3-gal; ↑78% que-3-glu; ↑59% que-3-ara; ↑68% que-3-xyl; ↑61% que-3-rha; *↑75% T.FOLs* DHCs: ↑ 67% phloridzin; ↑ 51% phloretin-2-xyloglucoside; *↑63% T.DHCs* HCAs: ↑31% chlorogenic acid; ↑4% neochlorogenic acid; ↑5% cryptochlorogenic acid; ↑51% coumaroyl quinic acid; *↑29% T. HCAs* *400–500 MPa* F3OLs/PACs: ↑2–13% CA; ↓10–19% procyanidin B2; ≈trimers; ↑131–161% dimers; ↓4–14% EC; *≈T.F3OLs/PACs* FOLs: ↑24% que-3-rut (400 MPa); ↑10–44% que-3-gal; ↓35% que-3-glu (400 MPa); ↓7–21% que-3-ara; ↓7–25% que-3-xyl; ↓7–23% que-3-rha; *↑5–29% T.FOLs* DHCs: ↓15% phloridzin; ↓1–14% phloretin-2-xyloglucoside; *↓11–↑8% T.DHCs* HCAs: ↓14–17% chlorogenic acid; ↓8–13% neochlorogenic acid; ↓8–16% cryptochlorogenic acid; ↓3–7% coumaroyl quinic acid; *↓13–16% T. HCAs*	-	Differences highly influenced by apple origin ↓Oligomers PACs → epimerization changes and depolymerization ↑FOLs: enhanced extraction by higher permeability or disruption of cell walls ↓FOLs: residual enzyme activity (PPO and POD) → oxidation	[12]
Apples (Spain)	HPP: 400 MPa-/5 min at 35 °C	No	F3OLs/PACs: ≈procyanidin B1, EC trimers and tetramers; ↓4% EC; ↑10% CA, ↑4% procyanidin B2; ↑65% EC-dimer; *↑3% T.F3OLs/PACs* FOLs: ↑9% que-3-rut; ↑35% que-3-gal; ↑22% que-3-glu; ↑30% que-3-ara; ↑32% que-3-xyl; ↑33% que-3-rha; *↑30% T.FOLs* DHCs: ≈phloridzin; ↓9% phloretin-2-xyloglucoside; *≈T.DHCs* HCAs: ↓13% chlorogenic acid; ↓9% neochlorogenic acid; ↓10% cryptochlorogenic acid; ↓17% coumaroyl quinic acid; *↓12% T. HCAs*		↓Oligomers PACs → epimerization changes and depolymerization	[53]
Cloudy apple juice	HPP: 600 MPa/5 minat 25 °C	12 weeks 4 ºC	F3OLs/PACs: ≈ CA; ↓13% EC; ↓45% procyanidin B1; *↓15% T. F3OLs/PACs* DHCs: ↓18% phloridzin HCAs: ≈ chlorogenic acid	F3OLs/PACs: ↓92% procyanidin B1; ↓61%; ↓15% CA DHCs: ↓71% phloridzin HCAs: ↓53% chlorogenic acid	↓F3OLs/PACs: residual PPO and POD activity → oxidation ↓DHCs: residual enzyme activity → oxidation	[54]

↑: increment vrs. control; ↓: diminution vrs. control; ≈: unchanged vrs. control; ∅: absence; O_2_: oxygen; ∑: sum total; Ctrl: control; TT: thermal treatment; HPP: high-pressure processing; F3OLs/PACs: proanthocyanidins; HCAs: hydroxycinnamics; DHCs: dihydrochalcones; FOLs: flavonols: T.: total; CA: catechin; EC: epicatechin: ECG: epicatechin gallate; EGC: epigallocatechin; que: quercetin; gal: galactoside; hex: hexoside; xyl; xyloside; rha: rhamnoside; pen: pentoside; ara: arabinoside; PPO: polyphenol oxidase; POD: peroxidase; ^a^ Total percentage of change (in italic) was calculated by adding the concentration of all polyphenols from the same family and comparing it before and after processing. These data are represented in Figure 3 and Figure 4. Control samples were the same matrixes (juices, puree, etc.) just before TT and HPP; ^b^ To study storage effects, samples after TT or HPPP at time 0 were used as control; ^c^ Control sample was the fresh fruit; ^d^ Studies without concentration data to calculate the percentage of change (kinetics or graphs).

**Table 3:** Impact of TT and HPP treatments on different apple and strawberry phenolic compounds. (F3OLs/PAC) flavan-3-ols + proanthocyanidins; (ATs) anthocyanins; (EA) ellagic acid; (FOLs) flavonols; (DHCs) dihydrochalcones; (HCAs) hydroxycinnamic acids.

	TT		HPP	
**F3OLs/** **PACs**	**8 studies/30 trials**		**6 studies/28 trials**	
	**Positive (10)**	**Negative (20)**	**Positive (13)**	**Negative (15)**
	Apple puree/juice Pasteurized strawberries Strawberry pulp/puree	Apple sauce/juice Strawberry puree	Pressurized apples Cloudy apple juice Strawberry puree	Strawberry puree/pulp Pressurized apples Apple juice
**ATs**	**7 studies/23 trials**		**6 studies/40 trials**	
	**Positive (5)**	**Negative (18)**	**Positive (16)**	**Negative (24)**
	Strawberry puree	Pasteurized strawberries Strawberry pulp/puree	Strawberry pulp/puree	Strawberry pulp/puree
**EA**	**5 studies/10 trials**		**3 studies/27 trials**	
	**Positive (6)**	**Negative (4)**	**Positive (13)**	**Negative (14)**
	Strawberry puree/pulp Pasteurized strawberries	Strawberry puree	Strawberry puree	Strawberry pulp
**FOLs**	**3 studies/17 trials**		**3 studies/11 trials**	
	**Positive (14)**	**Negative (3)**	**Positive (9)**	**Negative (2)**
	Applesauce Apple juice	Applesauce Apple juice	Pressurized apples Apple juice	Pressurized apples
**DHCs**	**3 studies/16 trials**		**6 studies/12 trials**	
	**Positive (12)**	**Negative (4)**	**Positive (6)**	**Negative (6)**
	Applesauce Apple juice	Applesauce Apple juice	Pressurized apples Apple puree	Pressurized apples Apple juice
**HCAs**	**4 studies/19 trials**		**4 studies/12 trials**	
	**Positive (5)**	**Negative (14)**	**Positive (5)**	**Negative (7)**
	Apple puree/juice	Applesauce/puree Apple juice	Apple juice	Pressurized apples Apple juice

F3OLs/PACs: proanthocyanidins; ATs: anthocyanins; EA: ellagic acid; FOLs: flavonols; DHCs: dihydrochalcones; HCAs: hydroxycinnamics.

## Data Availability

Data sharing is not applicable to this article.
